# Impact of glucocorticoids on the efficacy of neoadjuvant chemoradiotherapy and survival of patients with locally advanced rectal cancer: a retrospective study

**DOI:** 10.1186/s12885-023-10592-0

**Published:** 2023-03-14

**Authors:** Xiaoxue Huang, Zhiyuan Zheng, Bangwei Zeng, Han Xiao, Hao Zheng, Zhuangbin Lin, Jianyuan Song, Anchuan Li, Pan Chi, Yinghong Yang, Benhua Xu, Rong Zheng

**Affiliations:** 1 Department of Radiation Oncology, Fujian Medical University Union Hospital, No.29 XinQuan Road, Gulou District, Fuzhou, Fujian 350001 People’s Republic of China; 2 Medical Technology and Engineering College of Fujian Medical University, Fuzhou, Fujian 350001 People’s Republic of China; 3 Nosocomial Infection Control Branch, Fujian Medical University Union Hospital, Fuzhou, Fujian 350001 People’s Republic of China; 4 Department of Pathology, Fujian Medical University Union Hospital, Fuzhou, Fujian 350001 People’s Republic of China; 5 Department of Medical Imaging Technology, College of Medical Technology and Engineering, Fujian Medical University, Fuzhou, Fujian Province People’s Republic of China; 6 Fujian Medical University Union Clinical Medicine College, Fujian Medical University, Fuzhou, Fujian Province People’s Republic of China; 7 School of Clinical Medicine, Fujian Medical University, Fuzhou, Fujian Province People’s Republic of China; 8 Department of Colorectal Surgery, Fujian Medical University Union Hospital, Fuzhou, Fujian 350001 People’s Republic of China; 9 Fujian Key Laboratory of Intelligent Imaging and Precision Radiotherapy for Tumors (Fujian Medical University), Fuzhou, Fujian Province People’s Republic of China; 10 Clinical Research Center for Radiology and Radiotherapy of Fujian Province (Digestive,Hematological and Breast Malignancies), Fuzhou, Fujian Province People’s Republic of China

**Keywords:** Rectal cancer, Glucocorticoids, Neoadjuvant chemoradiotherapy

## Abstract

**Background:**

Preclinical studies suggest that glucocorticoids (GCs) promote the proliferation and development of colorectal cancer. Because GCs are broadly prescribed for treatment-related adverse events in patients with locally advanced rectal cancer (LARC) receiving neoadjuvant chemoradiotherapy (NCRT), it’s essential to assess the effect of GCs on clinical outcomes.

**Methods:**

LARC cases treated with NCRT followed by surgery were assessed retrospectively. Evaluation of the relationship between GCs use (GCs vs. non-GCs) and neoadjuvant rectal (NAR) score (as a three-level categorical dependent variable) was performed using multivariable multinomial logistic regression (MLR). We also examined the relationship between the accumulated dose of GCs and NAR using multivariate MLR. Survival analysis of disease-free survival (DFS) and overall survival (OS) was performed using the Kaplan–Meier method. Multivariate Cox regression was used to assess confounding factors that could influence OS and DFS.

**Results:**

This retrospective cohort study included 790 patients with newly diagnosed non-metastatic LARC (T3-4/N + M0) who received NCRT followed by surgery between January 2012 and April 2017. The end of the follow-up period was May 11, 2022. Among the 790 patients with LARC, 342 (43.2%) received GCs treatment and 448 (56.8%) did not during the NCRT-to-surgery period. GCs medication was significantly different between mid-NAR (8–16) and low-NAR (< 8) (odds ratio [OR], 0.615; 95% CI, 0.420–0.901; *P* = 0.013), and the high-NAR (> 16) and low-NAR (0.563; 0.352–0.900; 0.016). Patients exposed to GCs, had a decreased 5-year OS (GCs vs. non-GCs = 80.01% (95% CI, 75.87%–84.37%) vs. 85.30% (82.06%–88.67%), *P* = 0.023) and poorer 5-year DFS (73.99% (69.45%–78.82%) vs. 78.7% (75.14%–82.78%), *P* = 0.045). The accumulated dose of GCs was an independent risk factor for OS (hazard ratio [HR], 1.007 [1.001–1.014], 0.036) and DFS (1.010 [1.004–1.017], 0.001).

**Conclusions and relevance:**

Our study revealed that GCs were associated with reduced efficacy of NCRT and worse clinical outcomes in patients with LARC during the NCRT-to-surgery period.

**Supplementary Information:**

The online version contains supplementary material available at 10.1186/s12885-023-10592-0.

## Introduction

Glucocorticoids (GCs) are used widely in non-hematologic cancer as supportive care comedication to alleviate the side effects induced by standard therapies due to their antiemetic, anti-inflammatory, and energy/appetite-stimulating effects [1]. However, concerns regarding the safety of GCs in patients with cancer have been expressed repeatedly [2]. Some studies have reported that the inhibition of tumor immunity and apoptosis by corticosteroids may promote tumor proliferation, leading to treatment resistance and possibly disease progression [3–8].

Among patients with locally advanced rectal cancer (LARC), neoadjuvant chemoradiotherapy (NCRT) is recommended before surgery, often leading to the prescription of GCs for adverse effect induced by combined cytotoxicity of drugs and irradiation. In our institution, GCs are frequently used to increase appetite, reduce weight loss, eliminate fatigue, and prevent vomite to manage corresponding treatment-related toxicities in clinical oncology [9–11]. Especially during NCRT, glucocorticoids are often administered for short-term use for their classical anti-inflammatory activities to ease the acute inflammatory damage of the colon and rectal tissue [12]. However, the clinical implications of NCRT and systemic corticosteroids therapy remain unclear. Therefore, this study aimed to evaluate the potential impact of GCs on the efficacy of NCRT and the long-term outcomes of patients with LARC.

## Methods

### Patient cohort

**Fig. 1:**
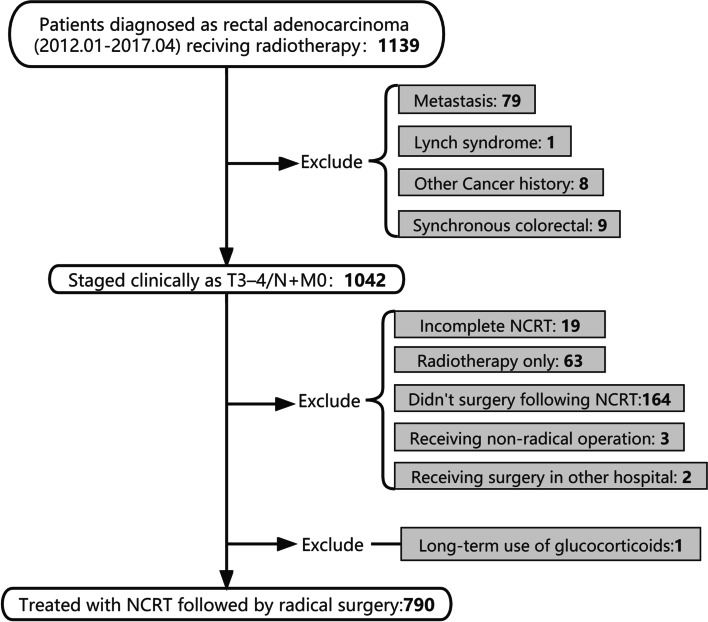
Flowchart of participant recruitment

Between January 2012 and April 2017, this retrospective, single-center, study reviewed patients diagnosed with LARC (T3-4/N +), pathologically confirmed as adenocarcinoma without distant metastasis, who were treated with NCRT followed by surgery. The study was approved by the institutional review board and the need for informed consent was waived owing to the retrospective nature of the study. This study followed the Strengthening the Reporting of Observational Studies in Epidemiology (STROBE) reporting guidelines. Patients were excluded if they required long-term GCs medication or were treated with other neoadjuvant therapy regimens such as radiation therapy alone, short-course radiation and combination with targeted therapy (Fig. 1).

### Data collection

The use of GCs during the period from the NCRT to surgery (NCRT-to-S) was determined by reviewing the patients’ pharmacy records. Information regarding the type of GCs, medication time, accumulated dose, and route of administration was collected. The accumulated dose of GCs during NCRT-to-S was expressed as total milligrams of dexamethasone equivalents.

Clinical data were collected for all patients, including age, sex, clinical TNM stage (cTNM), tumor location, baseline levels of carcinoembryonic antigen (CEA) and carbohydrate antigen 19–9 (CA199), received chemotherapy before NCRT, concurrent chemotherapy (CCT) regimen, radiotherapy technology, number of lymph nodes dissected, received postoperative chemotherapy, and yield pathological TNM stage (ypTNM).

### Therapeutic schemes

Neoadjuvant chemoradiotherapy for patients with LARC as preoperative treatment consist of concurrent chemoradiotherapy (CCRT) plus neoadjuvant chemotherapy in our institution. Most patients received consolidated chemotherapy followed by CCRT, while few received preoperative chemotherapy before CCRT, both of which are supported by the National Comprehensive Cancer Network [13]. All patients underwent curative total mesorectal excision (TME) and pelvic node sampling or dissection- 6–8 weeks after preoperative NCRT.

Gross tumor volume (GTV) was contoured based on digital rectal examination, endoscopic ultrasound and abdominopelvic MRI. The clinical target volume (CTV) included a minimum of 3 cm craniocaudal margin to the GTV and the entire mesorectum and presacral and internal iliac lymph node drainage regions. Planning target volumes (PTVs) for GTV and CTV were generated with an additional 10 mm margin separately. RT was applied to the PTV-CTV with 45 Gy in 25 fractions, with or without a simultaneous integrated boost to the PTV-GTV of 50 Gy/25fractions.

The patients received capecitabine-based or 5-fluorouracil (5-FU) based concurrent chemotherapy regimens in preoperative and postoperative chemotherapy. The four chemotherapeutic regimens with dosages were given as follows: (1) xeloda alone: xeloda 825 mg/m2 twice daily oral, during the whole period of radiotherapy; another 1 cycle increased dosages to 1250 mg/m2 was performed in 2 weeks during the waiting period. (2) XELOX: oxaliplatin 130 mg/m2 intravenously guttae day 1, xeloda 825 mg/m2 twice daily oral days 1–14, every 3 weeks, for 2 cycles during concurrent radiotherapy; another 2 cycles were performed during the interval from the end of radiation to surgery; (3) FOLFOX4: oxaliplatin 85 mg/m2 intravenously guttae day 1, leucovorin 200 mg/m2 intravenously guttae day 1 and day 2, 5-FU 400 mg/m2 intravenously bolus on day 1,followed by 600 mg/m2/day × 2 days continuous infusion, every 2 weeks to a total of 6 month preoperative therapy. (4) De Gramont: leucovorin 200 mg/m2 intravenously guttae day 1 and day 2, 5-FU 400 mg/m2 intravenously bolus on day 1, followed by 600 mg/m2/day × 2 days continuous infusion, every 2 weeks to a total of 6 months preoperative therapy.

### Outcome measures

The primary outcome of this study was overall survival (OS), and secondary end points included disease-free survival (DFS), neoadjuvant rectal (NAR) score, yield pathological complete response (ypCR), and tumor regression grade (TRG). OS was defined as the time from diagnosis to death due to any cause. DFS was defined as the time from diagnosis to the first event (tumor progression or death from any cause). The NAR score was calculated using the following formula: NAR = [5 × ypN—3 × (cT-ypT) + 12]^^2^ / 9.61 [14], where cT = 3–4, pT = 0–4, and pN = 0–2. According to NSABP R-04 data, NAR was further categorized into three groups: low-NAR (NAR < 8), mid-NAR (NAR = 8–16), and high-NAR (NAR > 16) [15]. TRG was graded according to the four-tier AJCC TRG classification [16]. And ypCR was defined as ypT_0_N_0_M_0_.

### Statistical analysis

Statistical significance was evaluated using Fisher’s exact test or χ2 test to compare categorical data, and the Mann–whitney U test was used to compare continuoues data of abnormal distribution between the two study groups. Evaluation of the relationship between GCs use (GCs vs. non-GCs) and NAR (as a three-level categorical dependent variable) was performed using multivariable multinomial logistic regression (MLR) adjusted for demographic, clinical, and treatment-related characteristics. The adjusted multivariable MLR analysis was firstly conducted using low-NAR as the reference group, which generated comparisons of mid-NAR vs. low-NAR, and high-NAR vs. low-NAR. A second MLR was conducted with mid-NAR as the reference group to yield comparisons of high-NAR vs. mid-NAR. We also examined the relationship between the accumulated dose of GCs and three-level NAR using multivariate MLR. Univariable Cox regression was used to evaluate the association of each variable with OS and DFS. We first taking all clinical pathological characteristics into regression model due to most of variables linking to treatment. Then, we used two methods to select important covariates, including using the stepwise forward procedure based on the likelihood ratio statistic and choosing terms with *p*-value < 0.1 in univariable Cox regression. For each covariate, the proportional hazards (PH) assumption was tested using statistical tests and graphical diagnostics based on the scaled Schoenfeld residuals by the functions cox.zph() and ggcoxzph() in the survival R package (version 3.2–13). In addition, it performs a global test for the model as a whole. To check the linear form of the accumulated dose of GCs as a continuous covariate, we used the function ggcoxfunctional() in the survminer R package (version 0.4.9), displaying graphs of the accumulated dose of GCs against martingale residuals of the null cox proportional hazards model. The survivalROC R package (version 1.0.3) was used to determine the best cutoff value for the accumulated dose of GCs associated with the 5-year OS of all patients. Survival analysis of DFS and OS was performed using the Kaplan–Meier method. The survival and forest plots were draw using the R package survminer (version 0.4.9) and forestplot (version 1.10.1), respectively. Two-tailed *P* values < 0.05 were considered statistically significant. Statistical analyses were performed using SPSS version 25.0, and all R packages mentioned were performed in R version 4.1.2.

## Results

### Characteristic of all patients

A total of 790 patients (502 [63.5%] men; 288 [36.5%] women; median [interquartile range] age, 56 [49–63] years) were included in the study according to the inclusion and exclusion criteria in our study. For the entire study population, the end of the follow-up period was May 11, 2022. The median (interquartile range, IQR) follow-up time was 84.30 (72.03–99.93) months.

Among them, 342 patients (43.2%) received GCs treatment during NCRT-to-S and 448 patients (56.8%) did not. The detailed baseline characteristics of GCs medication are presented in Table 1 (placed at the end of the document text file) (i.e., GCs vs. non-GCs). Overall, data on sex, CA199, tumor location, clinical stage, and days of radiotherapy appeared to be balanced. Patients exposed to GCs were significantly younger (age, median [IQR], GCs vs. non-GCs, 55 [48–62.25] vs. 57 [49–64], *P* = 0.050), had higher CEA level (CEA, 4.7 [2.30–11.90] vs. 3.70 [2.00–8.03], *P* = 0.013) and shorter interval between radiotherapy and surgery (days, 61 [55–66] vs. 64 [60–71], *P* < 0.001). In addition, compared with the non-GCs group, the intensity of anti-cancer treatment appeared to be higher in patients in GCs group. Receiving chemotherapy before NCRT (55 [16.1%] vs. 15 [3.3%], *P* < 0.001),multi-drug CCT regimens (Xeloda (151 [44.2%] vs. 398 [88.8%]), XELOX (137 [40.1%] vs. 43 [9.6%]), FOLFOX4 (35 [10.2%] vs. 3 [0.7%], De Gramont (19 [5.6%] vs. 4 [0.9%]), *P* < 0.001), and 3-dimensional conventional radiotherapy (3D-CRT) (123 [36.0%] vs. 79 [17.6%], *P* < 0.001) were more common among patients in the GCs group.

**Table 1:** Comparison of baseline characteristics in GCs group and non-GCs group

Characteristic	Patients, No. (%)		*P*
	**GCs (*n* = 342)**	**Non-GCs (*n* = 448)**	
Age, years, median (IQR)	55 (48–62.25)	57 (49–64)	0.050
Sex			0.802
Male	219 (64.0)	283 (63.2)	
Female	123 (36.0)	165 (36.8)	
CEA, ng/ml, median (IQR)	4.7 (2.30–11.90)	3.70 (2.00–8.03)	0.013
CA199, U/ml, median (IQR)	12.83 (6.89–22.55)	11.16 (6.09–19.91)	0.087
Tumor location			0.491
Low	161 (47.1)	192 (42.9)	
Middle	169 (49.4)	240 (53.6)	
High	12 (3.5)	16 (3.5)	
cT category			0.080
cT_1-3_	154 (45.0)	174 (38.8)	
cT_4_	188 (55.0)	274 (61.2)	
cN category			0.230
cN_0_	29 (8.5)	28 (6.3)	
cN_+_	308 (90.1)	415 (92.6)	
Missing	5 (1.5)	5 (1.1)	
Chemotherapy before NCRT			< 0.001
Yes	55 (16.1)	15 (3.3)	
No	287 (83.9)	433 (96.7)	
CCT regimen			< 0.001
Xeloda	151 (44.2)	398 (88.8)	
XELOX	137 (40.1)	43 (9.6)	
FOLFOX4	35 (10.2)	3 (0.7)	
De Gramont	19 (5.6)	4 (0.9)	
SIB to GTV			< 0.001
Yes	281 (82.2)	435 (97.1)	
No	61 (17.8)	13 (2.9)	
Radiotherapy technology			< 0.001
IMRT	210 (61.4)	347 (77.5)	
3D-CRT	123 (36.0)	79 (17.6)	
VAMT	9 (2.6)	22 (4.9)	
Days of radiotherapy, median (IQR)	37 (35–39)	37 (35–39)	0.849
Interval between radiotherapy and surgery, day, median (IQR)	61 (55–66)	64 (60–71)	< 0.001

*Abbreviations*: *GCs* glucocorticoids, *IQR* interquartile range, *CEA* carcinoembryonic antigen, *CA19-9* carbohydrate antigen 19–9, *cT category* clinical tumor category, *cN category* clinical lymph-node category, *NCRT* neoadjuvant chemoradiotherapy, *CCT* concurrent chemotherapy, *SIB* simultaneous integrated boost, *GTV* gross tumor volume, *IMRT* intensity modulated radiation therapy, *3D-CRT* 3-dimensional conventional radiotherapy, *VAMT* volumetric modulated arc therapy, *TRG* tumor regression grade, *NAR* neoadjuvant rectal score

### Impact of GCs on the efficacy of NCRT

Assessing the association of GCs medication with short-term efficacy of NCRT, revealed no significant differences in ypCR (GCs vs. non-GCs, 72 [21.1%] vs 98 [21.9%], *P* = 0.780) and TRG (TRG_0_: 75 [21.9%] vs. 103 [23.0%], TRG_1_: 117 [34.2%] vs. 145 [32.4%], TRG_2_: 128 [37.4%] vs. 173 [38.6%], TRG_3_: 22 [6.4%] vs. 27 [6.0%]), *P* = 0.936) between the GCs and non-GCs groups. However, patients with GCs medication had higher NAR score (mean rank: 419.21 vs. 377.40, with the same median value [IQR]: 8.43 [3.75–14.98], *P* = 0.010), which indicate poor clinical outcomes.

Among the three comparisons of NAR levels in the adjusted multivariable MLR analysis, GCs medication significantly differed between mid-NAR and low-NAR (odds ratio [OR], 0.615; 95% confidence interval (CI), 0.420–0.901; *P* = 0.013), as well as high-NAR and low-NAR (0.563; 0.352–0.900; 0.016) (Table 2) (placed at the end of the document text file). This indicates that compared with a patient who did not receive GCs, a patient who did had 1.626 times higher odds (i.e., 1 ÷ 0.615) of being in the mid-NAR classification relative to the low-NAR classification and had 1.776 times higher odds (i.e., 1 ÷ 0.563) of being in the high-NAR classification relative to the low-NAR classification. However, the accumulated dose of GCs was close to being statistically significant only in distinguish high-NAR and low-NAR classification (1.01; 1.000–1.020; 0.055) (Additional file 1).

**Table 2:** The multinomial logistic regression analysis of NAR

Variable	Mid-NAR vs Low-NAR				High-NAR vs Low-NAR				High-NAR vs Mid-NAR			
	**OR**	**95% CL for OR**		***P***	**OR**	**95% CL for OR**		***P***	**OR**	**95% CL for OR**		***P***
		**Lower**	**Upper**			**Lower**	**Upper**			**Lower**	**Upper**	
Age	1.016	1.000	1.032	0.057	1.001	0.982	1.021	0.895	0.986	0.967	1.004	0.134
Sex (Female: Male)	0.738	0.524	1.038	0.081	0.710	0.463	1.087	0.115	0.962	0.625	1.480	0.860
CEA	1.011	1.002	1.020	**0.016**	1.008	0.999	1.017	0.078	0.997	0.994	1.001	0.143
CA199	1.002	0.999	1.005	0.125	1.003	1.000	1.006	**0.035**	1.001	0.999	1.003	0.276
Tumor Location												
High: Low	4.245	1.484	12.146	**0.007**	2.394	0.677	8.471	0.176	0.564	0.204	1.561	0.270
Middle: Low	1.416	1.008	1.990	**0.045**	1.225	0.805	1.863	0.344	0.865	0.566	1.320	0.500
Chemotherapy before NCRT (No: Yes)	1.030	0.434	2.446	0.946	2.479	0.723	8.504	0.149	2.407	0.698	8.296	0.164
CCT regimen												
Xelox: Xeloda	0.727	0.463	1.141	0.166	0.929	0.544	1.587	0.788	1.278	0.749	2.183	0.368
De Gramont:Xeloda	1.068	0.257	4.428	0.928	0.728	0.091	5.843	0.765	0.682	0.087	5.367	0.716
FOLFOX4: Xeloda	0.498	0.159	1.557	0.231	0.798	0.209	3.055	0.742	1.604	0.410	6.267	0.497
SIB to GTV (No: Yes)	1.114	0.429	2.892	0.825	0.867	0.274	2.743	0.808	0.779	0.257	2.362	0.659
Radiotherapy technology												
VAMT:3D-CRT	0.773	0.309	1.937	0.583	0.508	0.148	1.742	0.281	0.657	0.183	2.361	0.520
IMRT:3D-CRT	1.360	0.863	2.143	0.185	0.916	0.539	1.557	0.746	0.674	0.394	1.152	0.149
Days of radiotherapy	1.005	0.964	1.047	0.831	1.001	0.950	1.055	0.972	0.996	0.946	1.050	0.893
Interval between radiotherapy and surgery	0.997	0.982	1.011	0.645	0.991	0.972	1.011	0.370	0.995	0.975	1.014	0.586
Number of lymph nodes dissected	1.033	1.006	1.060	**0.017**	1.091	1.058	1.124	** < 0.001**	1.056	1.026	1.087	** < 0.001**
GCs use (No: Yes)	0.615	0.420	0.901	**0.013**	0.563	0.352	0.900	**0.016**	0.915	0.579	1.447	0.704

*Abbreviations*: *OR* odds ratio, *CL* confidence limits, *NAR* neoadjuvant rectal score, *CEA* carcinoembryonic antigen, *CA19-9* carbohydrate antigen 19–9, *NCRT* neoadjuvant chemoradiotherapy, *CCT* Concurrent chemotherapy, *SIB* simultaneous integrated boost, *GTV* gross tumor volume, *IMRT* intensity modulated radiation therapy, *3D-CRT* 3-dimensional conventional radiotherapy, *VAMT* volumetric modulated arc therapy, *GCs* glucocorticoid

### Impact of GCs on survival

**Fig. 2:**
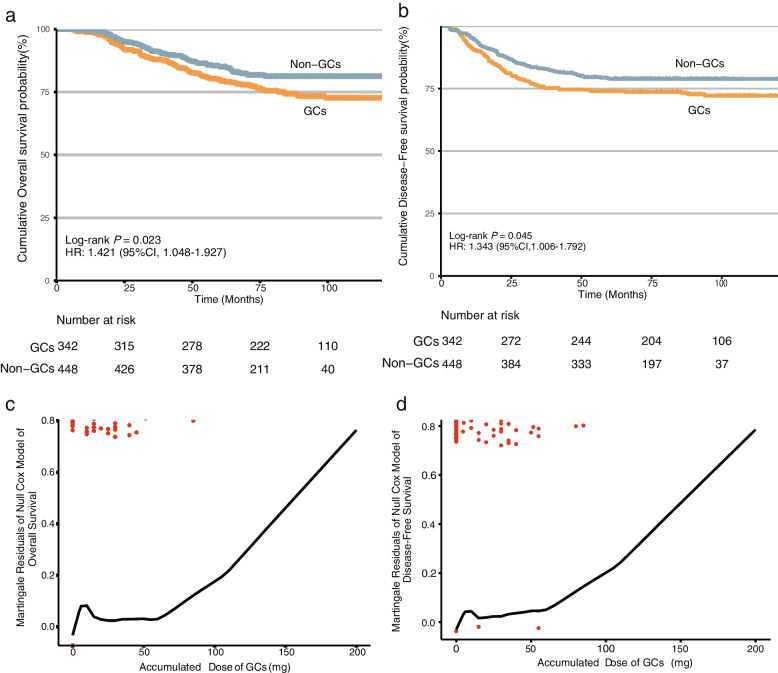
Survival analysis based on GCs medication. Kaplan–Meier curves of overall survival (**a**) and diseases-free Survival (**b**) by GCs medication. Check the linearity assumption of accumulated dose of GCs in the Cox proportional hazards model of overall survival (**c**) and diseases-free survival (**d**)

In patients exposed to GCs, we observed a decreased 5-year OS (GCs vs. non-GCs, 80.01% (95%CI, 75.87%–84.37%) vs. 85.30% (95%CI 82.06%–88.67%, *P* = 0.023) and a poorer 5-year DFS (GCs vs. non-GCs, 73.99% (95%CI, 69.45%–78.82%) vs. 78.7% (95%CI, 75.14%–82.78%), *P* = 0.045) compared with patients without GCs medication in Kaplan–Meier analysis (Fig. 2a-b). Besides, the accumulated dose of GCs as a continuous covariate was determined to have a linear form in both OS and DFS Cox regression models (Fig. 2c-d). Following univariable analysis to identify the potential predictors of DFS and OS (Additional files 2 and 3), GCs administration (hazard ratio [HR], 1.421 [95%CI, 1.048–1.927, *P* = 0.024) and accumulated dose of GCs (1.008 [1.000–1.011], 0.003) were associated with reduced OS, respectively, as was advanced advanced ypTNM stage (ypII vs. ypCR: 2.561 [1.475–4.447], 0.001; ypIII vs. ypCR: 4.649 [1.475–4.447], < 0.001; ypIV vs. ypCR: 14.291 [6.683–30.563], < 0.001). GCs administration (1.343 [1.006–1.792], 0.024), accumulated dose of GCs (1.009 [1.003–1.015], 0.002), and advanced ypTNM stage (ypII vs. ypCR: 3.913 [2.140–7.155], < 0.001; ypIII vs. ypCR: 6.985 [3.885–12.557], < 0.001; ypIV vs. ypCR: 44.39 [20.492–96.157], < 0.001) also significantly associated with diminished DFS.

**Fig. 3:**
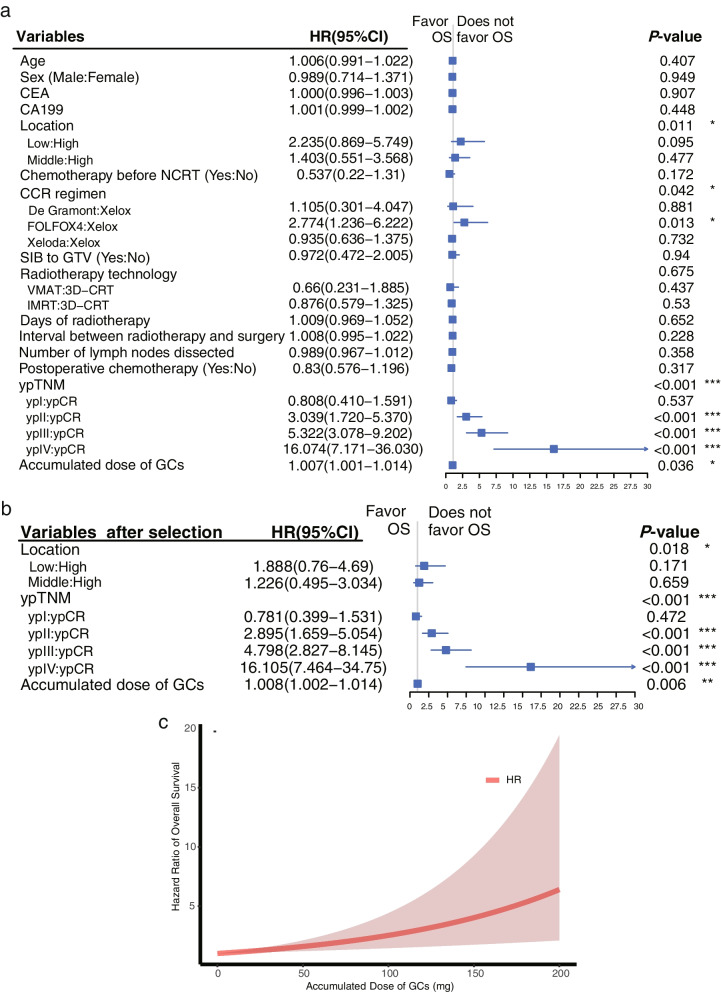
Multivariable Cox analysis of Overall Survival. **a** Multivariable Cox analysis of overall survival including all clinical pathological characteristics. **b** Multivariable Cox analysis of overall survival with the stepwise forward procedure of variables selection on the basis of the likelihood ratio statistic. **c** The graph of hazard rate of accumulated dose of GCs for death against its value adjusted by tumor location and ypTNM. Abbreviations: GCs, glucocorticoids; CEA, carcinoembryonic antigen; CA19-9, carbohydrate antigen 19–9; NCRT, neoadjuvant chemoradiotherapy; CCT, concurrent chemotherapy; SIB, simultaneous integrated boost; GTV, gross tumor volume; ypTNM, yield pathological tumor node metastasis stage

Further, we evaluated its impact on survival in a multivariable model covering age, sex, CEA, CA199, tumor location, chemotherapy before NCRT, CCR regimen, SIB to GTV, radiotherapy technology, days of radiotherapy, interval between radiotherapy and surgery, number of lymph nodes dissected, postoperative chemotherapy, and ypTNM, after the proportional hazards assumption for each covariate included in the OS and DFS Cox models was tested (Additional file 4, Figure S 1- 2). Following a multivariable Cox analysis of OS, higher accumulated dose of GCs (1.007 [1.001–1.014], *P* = 0.036) was associated with decreased OS as well as advanced ypTNM stage (ypII vs. ypCR: 3.039 [1.720–5.370], < 0.001; ypIII vs. ypCR: 5.322 [3.078–9.202], < 0.001; ypIV vs. ypCR: 16.074 [7.171–36.030], < 0.001), receiving FOLFOX4 regimen compared with XELOX as CCR (2.774 [1.236–6.222], 0.013) (Fig. 3a). After stepwise variable selection, a higher accumulated dose of GCs (1.008 [1.002–1.014], 0.006) remained associated with a worse OS (Fig. 3b). The estimated HR of the accumulated dose of GCs for death adjusted by tumor location and ypTNM increased with an increase in its level (Fig. 3c). The result were virtually unchanged from additional mutivariable Cox analysis of OS by hand-on selection of variables with *p*-value < 0.1 in univariable analysis of OS (Additional file 2).

**Fig. 4:**
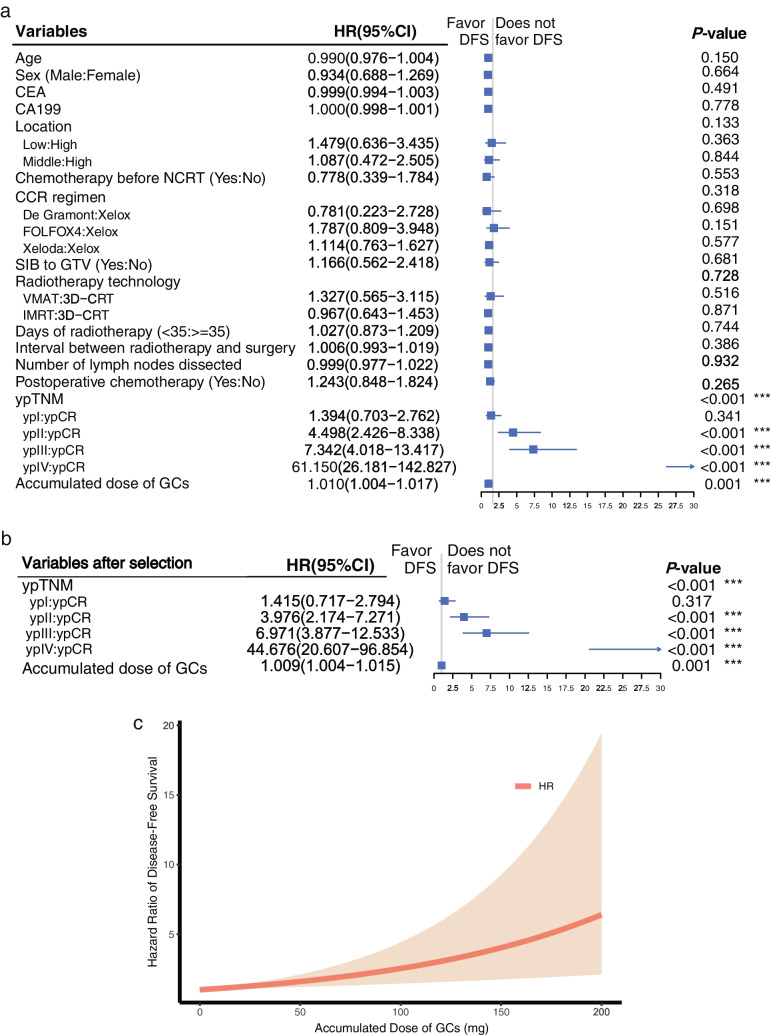
Multivariable cox analysis of diseases-free survival. **a** Multivariable Cox analysis of diseases-free survival including all clinical pathological characteristics. Days of radiotherapy was transformed as 2-class categorical variable as it didn’t satisfy proportional hazards assumption as continuous variable (Schoenfeld Individual Test, *P* = 0.046). **b** Multivariable Cox analysis of diseases-free survival with the stepwise forward procedure of variables selection on the basis of the likelihood ratio statistic. **c** The graph of hazard rate for of accumulated dose of GCs for diseases against its value adjusted by ypTNM. Abbreviations: GCs, glucocorticoids; CEA, carcinoembryonic antigen; CA19-9, carbohydrate antigen 19–9; NCRT, neoadjuvant chemoradiotherapy; CCT, concurrent chemotherapy; SIB, simultaneous integrated boost; GTV, gross tumor volume; ypTNM, yield pathological tumor node metastasis stage

Regarding multivariable Cox analysis of DFS, a higher accumulated dose of GCs (1.010 [1.004–1.017], 0.001) and advanced yield pathological stage (ypII vs. ypCR: 4.498 [2.426–8.338], < 0.001; ypIII vs. ypCR: 7.342 [4.018–13.417], < 0.001; ypIV vs. ypCR: 61.150 [26.181–142.827], < 0.001) were associated with diminished DFS (Fig. 4a). Moreover, a higher accumulated dose of GCs (1.009 [1.004–1.015], 0.001) was still a risk factor for DFS after variable selection (Fig. 4b), and the estimated HR of the accumulated dose of GCs for diseases adjusted by ypTNM risen with increasing accumulated dose of GCs (Fig. 4c). It was consistent with additional mutivariable Cox analysis of DFS including variables with *p*-value < 0.1 in univariable analysis of DFS (Additional file 3).

## Discussion

This retrospectively study reports that during the NCRT-to-S period, GCs medication is associated with decreased OS and DFS of LARC patients in a dose-dependent manner, and higher NAR score after adjusting for confounding factors in the multivariate analysis.

Corticosteroids, specifically GCs, are critical for the feedback inhibition of stress response and immune system homeostasis, by mediating potent anti-inflammatory and immunosuppressive effects. These effects can dampen autoimmune and inflammatory diseases, and may result in unintended consequences in cancer. It has been suggested that GCs regulate various immune cell activities. Innate and adaptive immunity to tumors is suppressed by corticosteroids, which inhibit the activity and proliferation of cytotoxic T cells and NK cells [17–19]. Dexamethasone alters the phenotype and function of dendritic cells (DCs) rendering them tolerogenic [20]. Corticosteroids are conducive to increasing the number of immunosuppressive regulatory T cells (Tregs) in the tumor microenvironment and, protecting tumor cells from immune recognition and attack [21, 22].

Several lines of evidence from the literature support the hypothesis that stress-induced and exogenous GCs cause the subversion of cancer therapy-elicited antineoplastic immune responses and subsequently abolish therapeutic control of tumors [3, 23], paticularly in immunotherapy [5, 6, 24, 25]. A retrospective analysis of 640 non-small cell lung cancer patients treated with anti-PD-(L)1 at the Sloan-Kettering Cancer Center and Guston-Roussy Cancer Center found that corticosteroid therapy was associated with reduced overall response rates and poor progression-free survival (HR = 1.3, *P* = 0.030) and overall survival (HR = 1.7, *P* < 0.001) [5].

In to colorectal cancer, immunoregulatory GCs also suppress anti-cancer immune responses. The synthesis and release of bioactive immunosuppressive corticosteroids have been proved to be one of the potential mechanisms of immune evasion of colorectal carcinoma [26]. Psychological distress is reported to induce the elevation of plasma corticosterone and expression of glucocorticoid-inducible factor Tsc22d3, which blocks type I interferon responses and subverts therapy-induced anticancer immunosurveillance in colorectal cancer and non–small cell lung carcinoma [23].

Moreover, GCs during the acute inflammatory phase have been shown to be detrimental to injury repair and tissue healing and increase the development of colorectal cancer. GCs administration can reduce intestinal inflammation, which, however, inversely delays tissue repair and accelerates the formation of aggressive colorectal cancers through the suppression of macrophage-mediated tissue repair and antitumor responses [27, 28]. Preadmission use of oral GCs increased the risk of 30-day mortality significantly [29] and the risk of anastomotic leakage modestly [30] after rectal cancer resection. In addition, preoperative dexamethasone administration has been reported to be associated with a raising rate of distant recurrence in patients undergoing colonic cancer resection [31]. These reports are consistent with our finding that receiving GCs treatment during NCRT reduces the effect of NCRT and worsens the long-term clinical outcomes. It may only repress intestinal inflammatory response without tissue repair during the acute inflammatory phase. Our data indicate that it is wise to manage adverse effects with other pharmacological agents with or without non-pharmacological methods during NCRT for LARC. These strategies could enable patients to avoid exposure to the untimely immunosuppression caused by corticosteroids.

GCs can promote tumor progression and therapeutic failure through indirect immunological effects and direct effects on malignant cells, which lies in GCs receptor (GR) signaling and genetic background. Upregulation of NF-κB and COX-2 may be involved in promoting the effect of GCs on the development of colorectal carcinoma [32] and glucocorticoid-GR-CDK1 signaling induces proliferation and invasion of colon cancer cells in mice [33]. Moreover, several small patient cohorts have revealed an association between GR expression and colorectal cancer progression. In 17 colorectal adenomas and paired normal mucosa, NR3C1 (a gene that encodes the GCs receptor (GR)) was reported as a key component of tumor formation [34]. In a cohort of 91 patients with colon cancer, GR expression was associated with tumor histopathological characteristics, proliferative capacity, cell cycle-related molecule expression, and survival [35]. High expression of GR in intestinal epithelial cells has been observed in colorectal cancer samples, which is associated with poor prognosis [27]. This indicates that different levels of GR expression and GC signaling in the tumor environment may be the underlying mechanisms of extraneous GCs in reducing the efficacy of NCRT. A deeper understanding of the causal relationship between GC signaling and the response to NCRT in vivo is needed to clinically manage toxicities caused by neoadjuvant therapy in LARC.

The NAR score is a prognostic evaluation model developed by the National Surgical Adjuvant Breast and Bowel Project (NSABP), the Radiation Therapy Oncology Group, and the Gynecologic Oncology Group (NRG) Oncology to serve as a short-term clinical trial surrogate endpoint on the basis of Valentini’s nomograms for OS [36]. The scoring formula incorporates a weighted combination of the cT, ypT, and ypN categories, representing a pseudo-continuous variable, with 24 possible discrete scores ranging from 0 to 100. NAR also reflects ypT downstaging, accounting for bulky or large tumors regressing but not to the degree of ypCR. It is particularly sensitive to changes in the factors affected by neoadjuvant therapy [15]. The prognostic role of the NAR score and its substitution for OS and DFS at the individual level has been validated in many large clinical trials [37, 38]. Although there was no significant correlation between GCs treatment and pCR or TRG, GCs is a risk factor for high-score NAR, which is consistent with the results of OS and DFS, suggesting that the influence of GCs may be slow and persistent.

A limitation of this study is the difficulty in determining whether GCs treatment is the cause of poor prognosis or a concomitant phenomenon of underlying mechanisms. We tried to make a preliminary judgment based on three aspects. Firstly, GCs treatment did not show a correlation with excessive tumor burden according to the balanced baseline data of baseline CA199 level, tumor location, and clinical stage between the GCs and non-GCs groups. Secondly, though patients with glucocorticoids have a higher portion of receiving chemotherapy before NCRT (induction therapy) than patients without. However, the patients with chemotherapy before NCRT only accounted for 8.86% of the whole cohort (6.96% in GCs group, 1.90% in Non-GCs group). And receiving induction chemotherapy before NCRT was neither the independent factor of overall survival nor diseases-free survival in our study. It consists with previous report that there are no differences in survival or local recurrence between induction chemotherapy before NCRT and consolidation chemotherapy after NCRT [39]. Thirdly, GCs treatment was more common in patients with a higher possibility to exposure to treatment toxicities, including chemotherapy before NCRT, receiving two-drug CCT more than a single-drug regimen, and using 3D-CRT more than IMRT, which could reduce acute and late gastrointestinal toxicity [40]. We can presume an impaired effect of acute gastrointestinal toxicity, especially acute radiation enteritis on survival upon GCs administration. However, acute radiation enteritis has been considered to be a good prognostic factor of clinical outcome according to some studies [41–43]. Hence, acute radiation enteritis as the common reason for GCs use may not cause poor outcome. Therefore, we should take a more cautious approach towards GCs use during NCRT for LARC.

Although we selected cases from 2012 to 2017 to reach sufficient follow-up time for the requirement of survival analysis, there are important limitations. In this period, several important clinical factors such as MSI testing, EMVI and CRM information haven’t been attached importance to and widely used in our institution. Hence, our study was unable to collect complete data on the factors mentioned above for further analysis. Prospective studies are warranted in the future. Additionally, owing to the lacking of data on why patients used GCs due to study’s retrospective nature, we can’t confirm whether the need for GCs to palliate symptoms among patients is a real poor prognostic factor, such as cancer-related pain. Further functional and mechanistic studies are needed to determine the persistent deleterious effects of corticosteroids in multivariate analyses. Whether GCs represent a correlation or causation, both patients and providers should be more cautious on the use of GCs during chemoradiotherapy for LARC.

## Conclusion

GCs medication is associated with reduced efficacy of NCRT and decreased long-term survival of patients with LARC during NCRT and waiting period for surgery. It is clinically relevant to recognize the effect of GCs on clinical outcomes for their more prudent use in the oncological practice of LARC. The underlying mechanisms and influence of GCs on the treatment of LARC can be explored in future studies, which can further elucidate standardized regimens for GCs.

## Supplementary Information

**Additional file 1: Table S1**.

**Additional file 2: Table S2**.

**Additional file 3: Table S3**.

**Additional file 4: Supplementary Figures**.

## Data Availability

The datasets used and/or analysed during the current study are available from the corresponding author on reasonable request.

## Abbreviations

GCs: Glucocorticoids
LARC: Locally advanced rectal cancer
NCRT: Neoadjuvant chemoradiotherapy
NAR: Neoadjuvant rectal score
MLR: Multinomial logistic regression
OS: Overall survival
DFS: Disease-free survival
STROBE: Strengthening the Reporting of Observational Studies in Epidemiology
NCRT-to-S: Neoadjuvant chemoradiotherapy to surgery
CEA: Carcinoembryonic antigen
CA199: Carbohydrate antigen 19–9
CCT: Concurrent chemotherapy
cTNM: Clinical tumour, lymph node, and metastasis stage
ypTNM: Yield pathological tumour, lymph node, and metastasis stage
GTV: Gross tumor volume
CTV: Clinical target volume
PTV: Planning target volume
TME: Total mesorectal excision
ypCR: Yield pathological complete response
TRG: Tumor regression grade
PH: Proportional hazards
IQR: Interquartile range
OR: Odds ratio
