# Supplementary material for: Impact of glucocorticoids on the efficacy of neoadjuvant chemoradiotherapy and survival of patients with locally advanced rectal cancer: a retrospective study
Source: BMC Cancer. 2023 Mar 14;23:238. doi: 10.1186/s12885-023-10592-0 (PMC10012496; doi:10.1186/s12885-023-10592-0)
Supplement: Supplementary file 1 — Additional file 1: Table S1. [file 12885_2023_10592_MOESM1_ESM.docx]

**TableS1 The multinomial logistic regression analysis of NAR**

| Variable | Mid-NAR vs Low-NAR | | | | High-NAR vs Low-NAR | | | | High-NAR vs Mid-NAR | | | |
| --- | --- | --- | --- | --- | --- | --- | --- | --- | --- | --- | --- | --- |
|  | **OR** | **95% CL for OR** | | ***P*** | **OR** | **95% CL for OR** | | ***P*** | **OR** | **95% CL for OR** | | ***P*** |
|  |  | Lower | Upper |  |  | Lower | Upper |  |  | Lower | Upper |  |
| Ages | 1.016 | 1.000 | 1.032 | 0.055 | 1.001 | 0.982 | 1.02 | 0.928 | 0.985 | 0.967 | 1.004 | 0.122 |
| CEA | 1.011 | 1.002 | 1.02 | **0.014** | 1.008 | 0.999 | 1.018 | 0.07 | 0.997 | 0.994 | 1.001 | 0.148 |
| CA199 | 1.002 | 0.999 | 1.005 | 0.114 | 1.003 | 1 | 1.006 | 0.034 | 1.001 | 0.999 | 1.003 | 0.294 |
| Days of radiotherapy | 1.005 | 0.964 | 1.048 | 0.816 | 0.999 | 0.948 | 1.052 | 0.962 | 0.994 | 0.943 | 1.047 | 0.815 |
| Interval between radiotherapy and surgery | 0.995 | 0.98 | 1.009 | 0.481 | 0.989 | 0.97 | 1.009 | 0.276 | 0.995 | 0.975 | 1.014 | 0.581 |
| Number of lymph nodes dissected | 1.033 | 1.007 | 1.061 | **0.015** | 1.091 | 1.058 | 1.125 | **<0.001** | 1.056 | 1.026 | 1.086 | **<0.001** |
| Sex (Female: Male) | 0.747 | 0.531 | 1.05 | 0.093 | 0.725 | 0.473 | 1.111 | 0.14 | 0.971 | 0.63 | 1.495 | 0.893 |
| Tumor Location | | | | | | | | | | | | |
| High: Low | 4.167 | 1.457 | 11.919 | **0.008** | 2.341 | 0.66 | 8.305 | 0.188 | 0.562 | 0.202 | 1.559 | 0.268 |
| Middle: Low | 1.381 | 0.985 | 1.936 | 0.061 | 1.195 | 0.786 | 1.815 | 0.404 | 0.865 | 0.567 | 1.32 | 0.501 |
| Chemotherapy before R (No: Yes) | 1.078 | 0.456 | 2.551 | 0.864 | 2.636 | 0.766 | 9.068 | 0.124 | 2.445 | 0.704 | 8.488 | 0.159 |
| Concurrent chemotherapy regimen | | | | | | | | | | | | |
| Xelox: Xeloda | 0.846 | 0.549 | 1.302 | 0.447 | 1.045 | 0.628 | 1.739 | 0.865 | 1.236 | 0.741 | 2.06 | 0.417 |
| De Gramont: Xeloda | 1.283 | 0.314 | 5.252 | 0.729 | 0.86 | 0.108 | 6.828 | 0.886 | 0.67 | 0.085 | 5.255 | 0.703 |
| FOLFOX4: Xeloda | 0.587 | 0.188 | 1.83 | 0.358 | 0.857 | 0.221 | 3.316 | 0.823 | 1.461 | 0.365 | 5.85 | 0.593 |
| SIB to GTV  (No: Yes) | 1.128 | 0.436 | 2.915 | 0.804 | 0.856 | 0.269 | 2.718 | 0.792 | 0.759 | 0.247 | 2.327 | 0.629 |
| Radiotherapy technology | | | | | | | | | | | | |
| VMAT:3D-CRT | 0.760 | 0.305 | 1.894 | 0.556 | 0.494 | 0.144 | 1.695 | 0.262 | 0.649 | 0.181 | 2.334 | 0.508 |
| IMRT:3D-CRT | 1.348 | 0.856 | 2.122 | 0.197 | 0.923 | 0.543 | 1.568 | 0.766 | 0.684 | 0.4 | 1.171 | 0.166 |
| Accumulated dose of GCs | 1.005 | 0.996 | 1.013 | 0.281 | 1.01 | 1.000 | 1.020 | 0.055 | 1.005 | 0.996 | 1.015 | 0.295 |

Abbreviations: OR, odds ratio; CL, confidence limits; NAR, neoadjuvant rectal score; CEA, carcinoembryonic antigen; CA19-9, carbohydrate antigen 19-9; NCRT, neoadjuvant chemoradiotherapy; CCT, Concurrent chemotherapy; SIB, simultaneous integrated boost; GTV, gross tumor volume; IMRT, intensity modulated radiation therapy; 3D-CRT, 3-dimensional conventional radiotherapy; VAMT, volumetric modulated arc therapy; GCs, glucocorticoids.
