# Supplementary material for: Impact of glucocorticoids on the efficacy of neoadjuvant chemoradiotherapy and survival of patients with locally advanced rectal cancer: a retrospective study
Source: BMC Cancer. 2023 Mar 14;23:238. doi: 10.1186/s12885-023-10592-0 (PMC10012496; doi:10.1186/s12885-023-10592-0)
Supplement: Supplementary file 2 — Additional file 2: Table S2. [file 12885_2023_10592_MOESM2_ESM.docx]

TableS2 The univariable and multivariable Cox regression analysis of OS

|  |  | Univariable analysis |  |  |  | Multivariable analysis |  |
| --- | --- | --- | --- | --- | --- | --- | --- |
| Variables | HR | 95% CL for HR | P |  | HR | 95% CL for HR | P |
| Age | 1.005 | 0.991-1.019 | 0.512 |  | / | / | / |
| Sex (Male:Female) | 1.029 | 0.750-1.414 | 0.858 |  | / | / | / |
| CEA | 1.000 | 0.998-1.003 | 0.853 |  | / | / | / |
| CA199 | 1.001 | 1.000-1.002 | 0.053 |  | 1.000 | 0.999-1.002 | 0.538 |
| Tumor Location |  |  | 0.279 |  | / | / | / |
| Low:High | 1.315 | 0.533-3.242 | 0.552 |  | / | / | / |
| Middle:High | 1.029 | 0.416-2.542 | 0.951 |  | / | / | / |
| Chemotherapy before NCRT (Yes:No) | 0.870 | 0.503-1.506 | 0.619 |  | / | / | / |
| CCT regimen |  |  | 0.353 |  | / | / | / |
| De Gramont:Xeloda | 0.827 | 0.328-2.088 | 0.688 |  | / | / | / |
| FOLFOX4: Xeloda | 1.366 | 0.720-2.590 | 0.340 |  | / | / | / |
| Xelox: Xeloda | 0.831 | 0.582-1.185 | 0.306 |  | / | / | / |
| SIB to GTV (Yes:No) | 0.800 | 0.501-1.278 | 0.350 |  | / | / | / |
| Radiotherapy technology |  |  | 0.167 |  | / | / | / |
| VAMT:3D-CRT | 0.448 | 0.162-1.238 | 0.122 |  | / | / | / |
| IMRT:3D-CRT | 0.789 | 0.567-1.098 | 0.161 |  | / | / | / |
| Days of radiotherapy | 1.008 | 0.971-1.044 | 0.637 |  | / | / | / |
| Interval between radiotherapy and surgery | 1.007 | 0.994-1.020 | 0.318 |  | / | / | / |
| Number of lymph nodes dissected | 1.008 | 0.988-1.028 | 0.439 |  | / | / | / |
| Postoperative chemotherapy (Yes:No) | 1.305 | 0.933-1.825 | 0.120 |  | / | / | / |
| ypTNM |  |  | <0.001 |  |  |  | <0.001 |
| ypI:ypCR | 0.788 | 0.402-1.544 | 0.487 |  | 0.786 | 0.401-1.541 | 0.484 |
| ypII:ypCR | 2.561 | 1.475-4.447 | 0.001 |  | 2.579 | 1.484-4.482 | 0.001 |
| ypIII:ypCR | 4.649 | 1.475-4.447 | <0.001 |  | 4.580 | 2.698-7.774 | <0.001 |
| ypIV:ypCR | 14.291 | 6.683-30.563 | <0.001 |  | 13.720 | 6.411-29.363 | <0.001 |
| GCs use (Yes:No:) | 1.421 | 1.048-1.927 | 0.024 |  | / | / | / |
| Accumulated dose of GCs | 1.008 | 1.000-1.011 | 0.003 |  | 1.008 | 1.002-1.014 | 0.005 |

Abbreviations: OS, overall survival; DFS, diseases-free survival; HR, hazard ratio; CL, confidence limits; CEA, carcinoembryonic antigen; CA19-9, carbohydrate antigen 19-9; NCRT, neoadjuvant chemoradiotherapy; CCT, Concurrent chemotherapy; SIB, simultaneous integrated boost; GTV, gross tumor volume; IMRT, intensity modulated radiation therapy; 3D-CRT, 3-dimensional conventional radiotherapy; VAMT, volumetric modulated arc therapy; GCs, glucocorticoids
