# Supplementary material for: Impact of glucocorticoids on the efficacy of neoadjuvant chemoradiotherapy and survival of patients with locally advanced rectal cancer: a retrospective study
Source: BMC Cancer. 2023 Mar 14;23:238. doi: 10.1186/s12885-023-10592-0 (PMC10012496; doi:10.1186/s12885-023-10592-0)
Supplement: Supplementary file 3 — Additional file 3: Table S3. [file 12885_2023_10592_MOESM3_ESM.docx]

**TableS3 The univariable and multivariable Cox regression analysis of DFS**

|  |  | **Univariable analysis** |  |  |  | **Multivariable analysis** |  |
| --- | --- | --- | --- | --- | --- | --- | --- |
| **Variables** | **HR** | **95% CL for HR** | **P** |  | **HR** | **95% CL for HR** | **P** |
| Age | 0.989 | 0.977-1.003 | 0.115 |  | / | / | / |
| Sex (Male:Female) | 0.996 | 0.857-1.157 | 0.959 |  | / | / | / |
| CEA | 1.000 | 0.997-1.003 | 0.893 |  | / | / | / |
| CA199 | 1.001 | 1.000-1.002 | 0.146 |  | / | / | / |
| Tumor Location |  |  | 0.711 |  | / | / | / |
| Low:High | 1.139 | 0.498-2.605 | 0.757 |  | / | / | / |
| Middle:High | 1.009 | 0.442-2.306 | 0.982 |  | / | / | / |
| Chemotherapy before NCRT (Yes:No) | 0.968 | 0.579-1.617 | 0.901 |  | / | / | / |
| CCT regimen |  |  | 0.590 |  | / | / | / |
| De Gramont:Xeloda | 1.076 | 0.458-2.524 | 0.867 |  | / | / | / |
| FOLFOX4: Xeloda | 1.351 | 0.714-2.558 | 0.355 |  | / | / | / |
| Xelox: Xeloda | 0.909 | 0.644-1.283 | 0.587 |  | / | / | / |
| SIB to GTV (Yes:No) | 0.828 | 0.520-1.318 | 0.427 |  | / | / | / |
| Radiotherapy technology |  |  | 0.653 |  | / | / | / |
| VAMT:3D-CRT | 0.816 | 0.317-1.797 | 0.614 |  | / | / | / |
| IMRT:3D-CRT | 0.864 | 0.625-1.194 | 0.376 |  | / | / | / |
| Days of radiotherapy | 0.996 | 0.963-1.030 | 0.817 |  | / | / | / |
| Interval between radiotherapy and surgery | 0.999 | 0.986-1.012 | 0.891 |  | / | / | / |
| Number of lymph nodes dissected | 1.025 | 1.005-1.044 | 0.012 |  | 1.003 | 0.982-1.024 | 0.802 |
| Postoperative chemotherapy (Yes:No) | 1.267 | 0.886-1.810 | 0.194 |  | / | / | / |
| ypTNM |  |  | **<0.001** |  |  |  | **<0.001** |
| ypI:ypCR | 1.427 | 0.723-2.817 | 0.306 |  | 1.411 | 0.715-2.787 | 0.321 |
| ypII:ypCR | 3.913 | 2.140-7.155 | **<0.001** |  | 3.954 | 2.159-7.242 | **<0.001** |
| ypIII:ypCR | 6.985 | 3.885-12.557 | **<0.001** |  | 6.885 | 3.799-12.479 | **<0.001** |
| ypIV:ypCR | 44.39 | 20.492-96.157 | **<0.001** |  | 44.428 | 20.468-96.435 | **<0.001** |
| GCs use (Yes:No:) | 1.343 | 1.006-1.792 | **0.045** |  | / | / | / |
| Accumulated dose of GCs | 1.009 | 1.003-1.015 | **0.002** |  | 1.009 | 1.004-1.015 | **0.001** |

Abbreviations: OS, overall survival; DFS, diseases-free survival; HR, hazard ratio; CL, confidence limits; CEA, carcinoembryonic antigen; CA19-9, carbohydrate antigen 19-9; NCRT, neoadjuvant chemoradiotherapy; CCT, Concurrent chemotherapy; SIB, simultaneous integrated boost; GTV, gross tumor volume; IMRT, intensity modulated radiation therapy; 3D-CRT, 3-dimensional conventional radiotherapy; VAMT, volumetric modulated arc therapy; GCs, glucocorticoid
