# Supplementary material for: Impact of glucocorticoids on the efficacy of neoadjuvant chemoradiotherapy and survival of patients with locally advanced rectal cancer: a retrospective study
Source: BMC Cancer. 2023 Mar 14;23:238. doi: 10.1186/s12885-023-10592-0 (PMC10012496; doi:10.1186/s12885-023-10592-0)
Supplement: Supplementary file 4 — Additional file 4: Supplementary Figures. [file 12885_2023_10592_MOESM4_ESM.docx]

**Figure S1 Test the proportional hazards assumption for each covariate included in overall survival Cox multivariable model fit.**


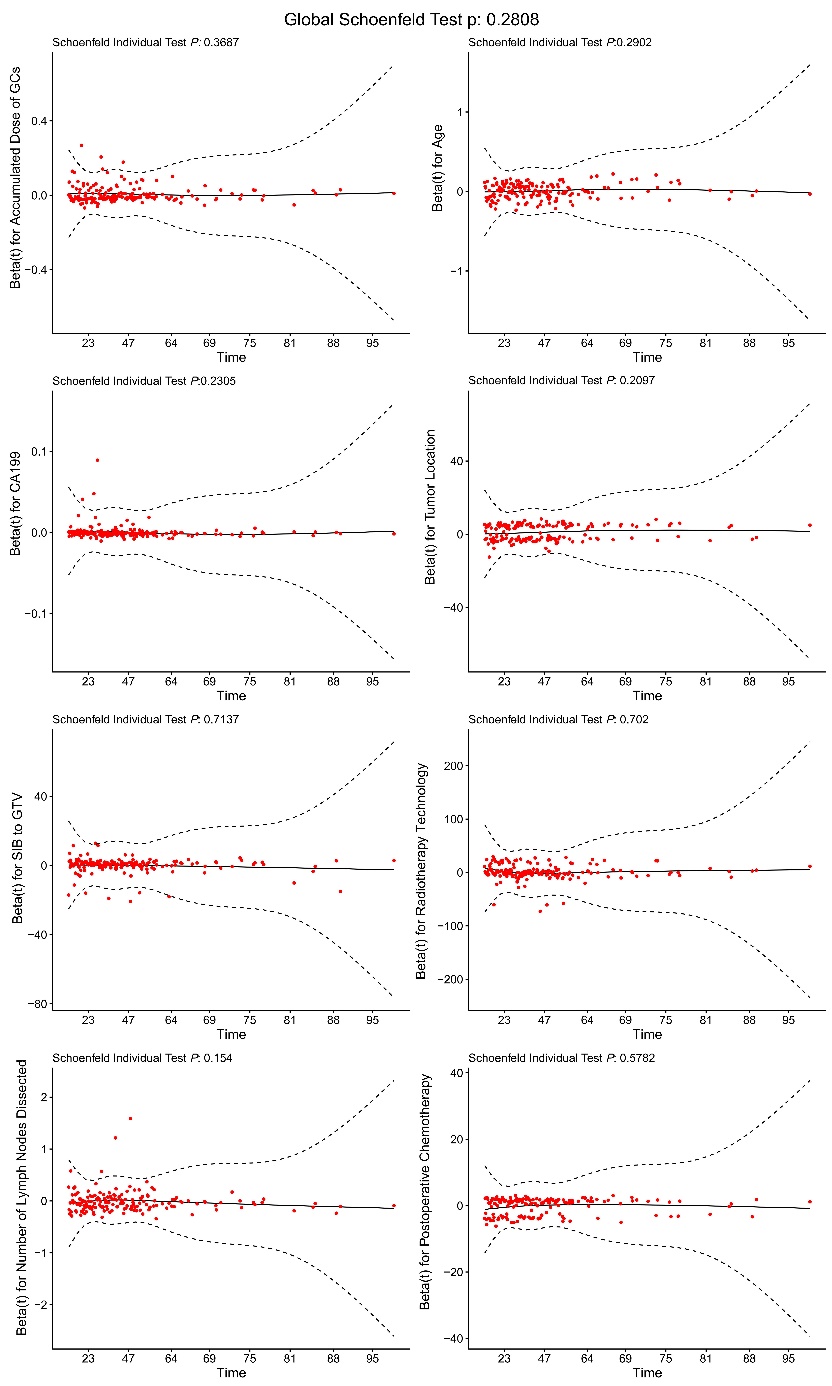

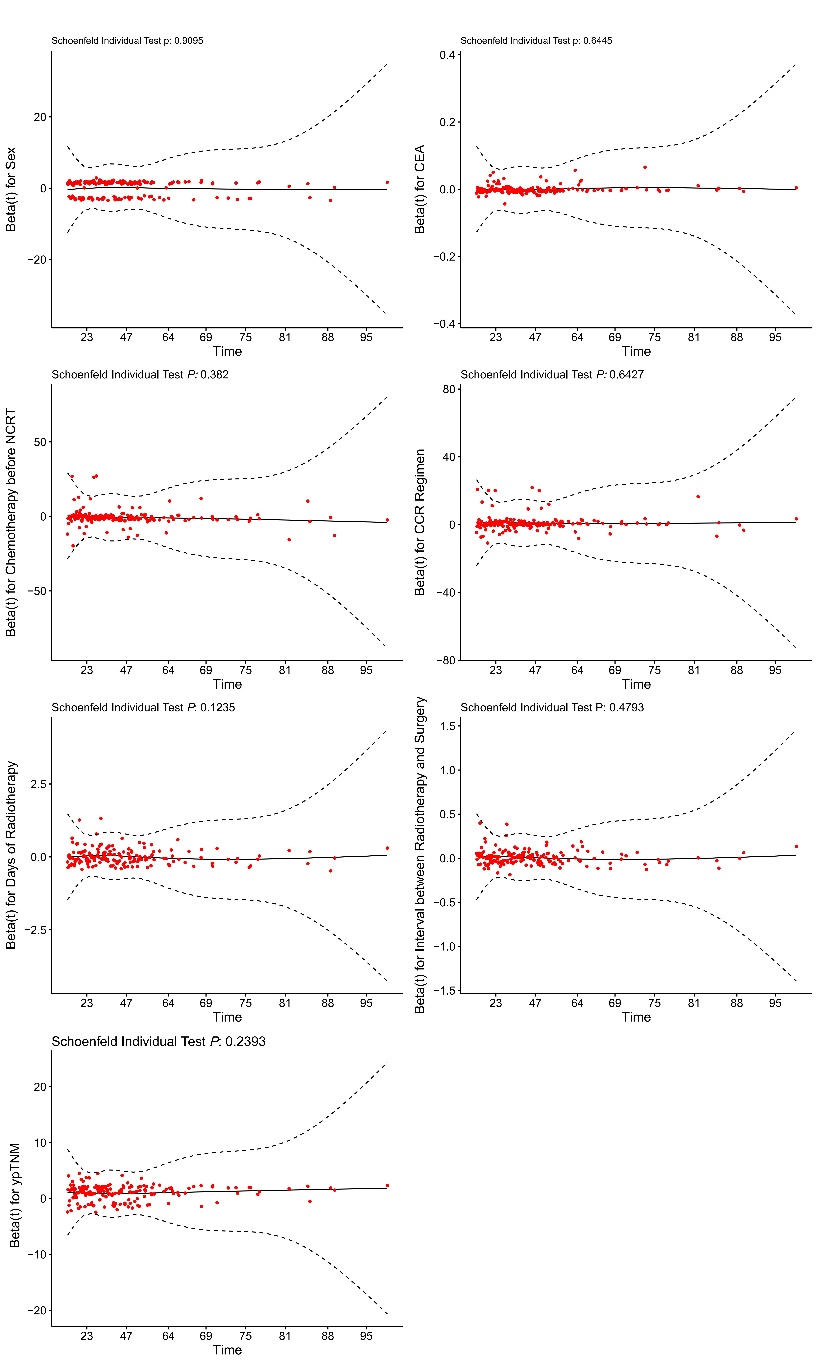


Graphical diagnostic of the scaled Schoenfeld residuals against the transformed time to test for

independence between residuals and time. Additionally, it performs a global test for the model as a

whole (*P*= 0.2808). Abbreviations: GCs, glucocorticoids; CEA, carcinoembryonic antigen; CA19-

9, carbohydrate antigen 19-9; NCRT, neoadjuvant chemoradiotherapy; CCT, concurrent

chemotherapy; SIB, simultaneous integrated boost; GTV, gross tumor volume; ypTNM, yield

pathological tumor node metastasis stage

|  |
| --- |

**Figure S2 Test the proportional hazards assumption for each covariate included in disease-free survival Cox multivariable model fit.**


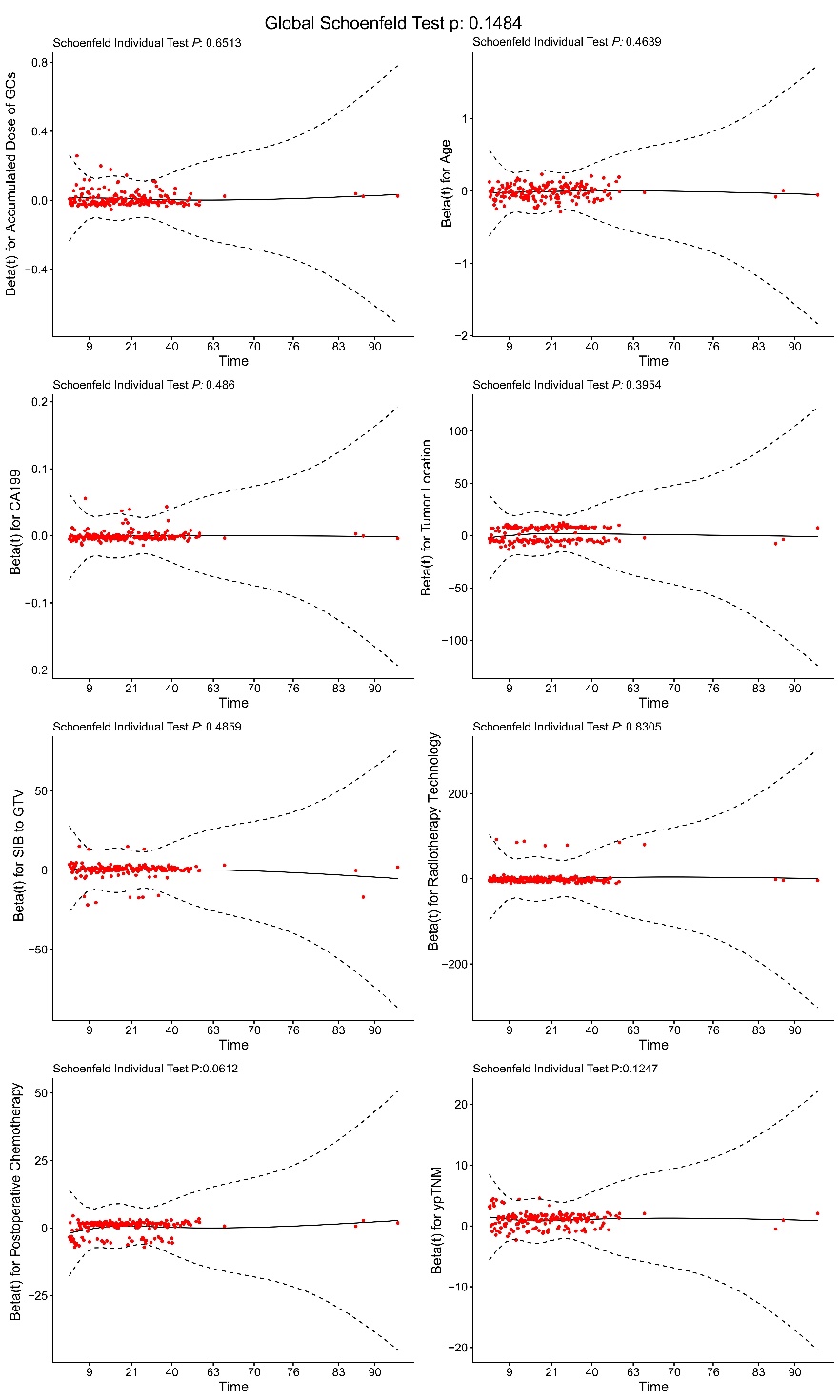

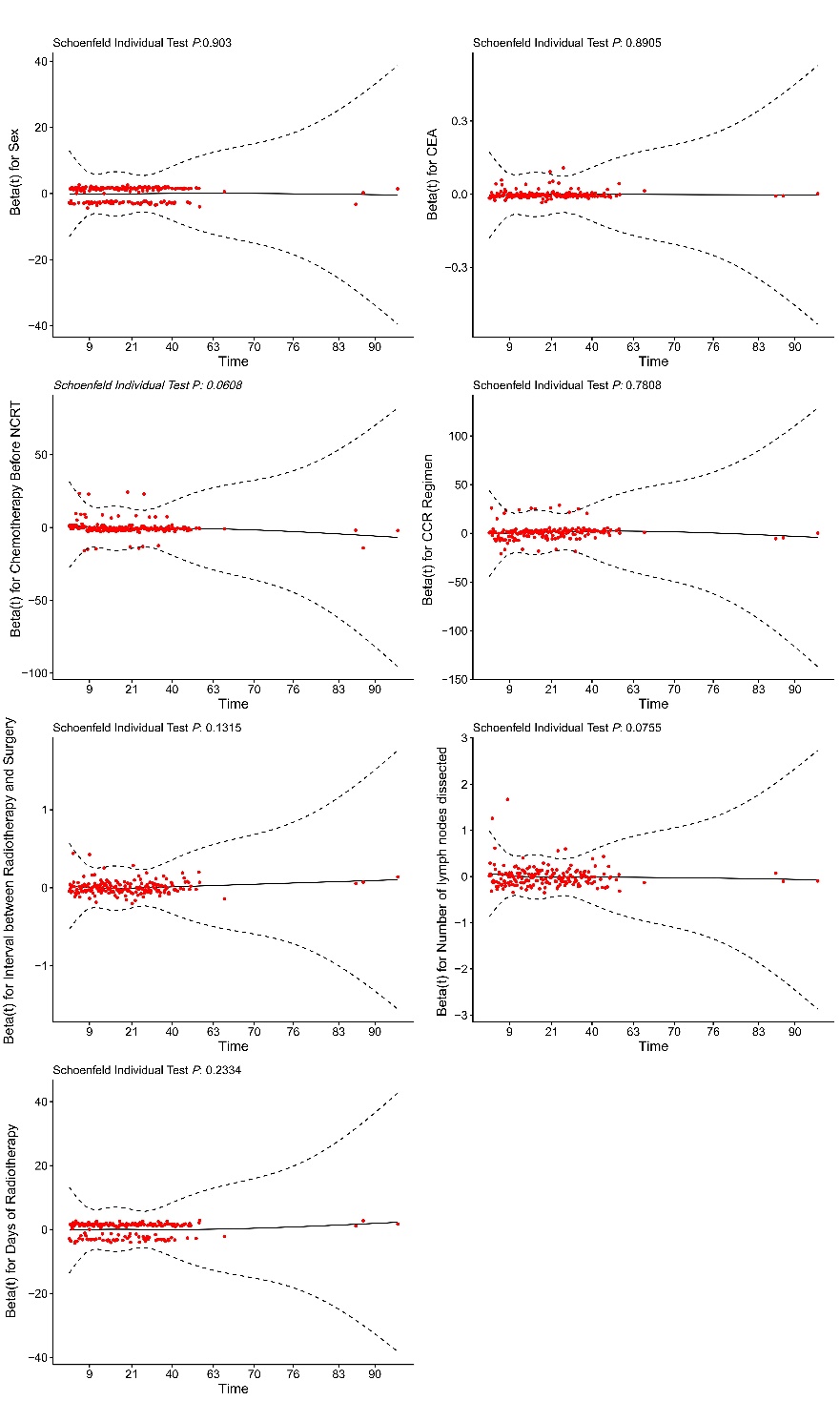


Graphical diagnostic of the scaled Schoenfeld residuals against the transformed time to test for independence between residuals and time. Additionally, it performs a global test for the model as a whole (*P*= 0.1484). Abbreviations: GCs, glucocorticoids; CEA, carcinoembryonic antigen; CA19-9, carbohydrate antigen 19-9; NCRT, neoadjuvant chemoradiotherapy; CCT, concurrent chemotherapy; SIB, simultaneous integrated boost; GTV, gross tumor volume; ypTNM, yield pathological tumor node metastasis stage.
